# 

**Published:** 1878-03

**Authors:** 


TO OUR SUBSCRIBERS.—With this number of the JOURNAL many subscriptions expire. We trust that our friends will promptly remit their renewals. Our bills for publishing the JOURNAL fall due monthly, and have to be paid. We think it is not asking too much to expect our subscribers to be alike prompt in paying for the JOURNAL. Many have written us, promising to pay at a given time, but the time has passed, and they have failed to comply with their promises. We cannot publish the JOURNAL without money. Remit by draft, P. O. money order, or registered letter, to H. H. DICKSON.

---

PAMPHLETS RECEIVED—*Compulsory Vaccination.*—The Establishment of a Uniform System of Vaccination for all Citizens and Inhabitants of the State of Louisiana, by Legislative enactment. By Joseph Jones, M.D., Professor of Chemistry and Clinical Medicine, Medical Department

University of Louisiana, etc., etc. From *The New Oleans Medical and Surgical Journal.* January, 1878.

On the Recognition and Management of the Gouty State in Diseases of the Skin. By L. Duncan Bulkley, A.M., M.D., Physician to the Skin Department, Demilt Dispensary, New York, etc. Reprint from the *American Practitioner.* November, 1877.

On the So-called Eczema Marginatum of Hebra (Tenia circinata cruris) as observed in America. A clinical study. By L. Duncan Bulkley, A.M., M.D., Physician to Skin Department Demilt Dispensary, etc., etc. Eczema and Psoriasis. By the same author.

The *Druggist & Chemist*, by C. C. Vanderbeck, M.D., Ph. D., Editor and Proprietor, is published by D. G. Brinton, M.D., at his Medical Publication Office, 115 South Seventh street, Philadelphia. This is a sprightly monthly of twenty pages, devoted to materia medica, pharmacy, chemistry and therapeutics, and sent to subscribers at $1.50 per year, in advance.

Studies in Pathological Anatomy. By Francis Delafield, M.D. This monthly contains five quarto pages of reading matter on pathological subjects, and three pathological plates each month, for $5.00 per annum.

*Braithwait's Retrospect, and Brinton's Half Yearly Compendium of Medical Science*, for January, have been received, and afford a treasure of recent medical literature.

---

### EDITORIAL CORRESPONDENCE.

### INDEX TO VOLUME XV.

Atlanta Academy of Medicine.. 7, 74, 159, 208, 403, 460, 548, 597, 677, 714
Arsenical Compounds, Ready Test for,............................ 14
Antiseptic Surgery,.................. 46
Atlanta Medical College,......46, 757
American Medical Association.. 50, 187
A Young Mother,...................... 55
Adulterated Medicine,.............. 71
Anæsthesia, the Discovery and Discoverers of,....................116
Antihydropin,.........................120
Anæsthesia by Ether in Young Subjects, Symptoms Resulting from,.................................126
Aneurisms of the Lower Extremity....................................179
American Medical College Association,........................189, 241
American Dermatological Association,.............................. 189
Arkansas Hot Springs,........... 222
Acute Rheumatism Treated with Salicylic Acid,............ 282
Acute Rheumatism, Salicylic Acid and Salicilate of Soda in,.................................... 687
Alcoholic Drinks, to Relieve Morbid Thist for................ 319
ANDERSON, R. B.,.................. 329
ADAMS, W. A.,...................... 335
Atmospheric Influences Controling Inebriety,............... 354
Asthma, Inhalation of Iodine in,.................................... 385
American Pharmaceutical Association,.......................... 432
Atlanta Medical College Clinic, 457, 590
Aspiration of the Perineam as a Means of Draining the Lower Part of the Pelvic Cavity,.............................. 572
A New Mucilage,.................. 580
ANDREWS, HENRY F.,.............. 577
Abnormal Condition of the Digestive Organs,.................. 593
Acute Tetanus following Wound in the Foot,....................... 605
Arm Presentation,................. 507
Action of Medicines,.............. 631
Atropine and Calabar in Eye Practice.......................... 639
American Pharmaceutical Association......................... 759
Antecedent Treatment of those exposed to Zymotic Diseases 750

BANKS, J. T.,........................ 4
Bronchitis,........................... 31
Best Means of Promoting Uinon by First Intention,.............. 105
Broncho-Pneumonitis,............ 329
Bromide of Potassium, the Use of Hypodermically,............ 335
Bromide of Lithium............... 448
BRAGG, SHIRLEY,................... 388
Botany in its Relations with Medicine.........393, 454, 523, 585
Bimanual, or Combined Turning,.................................. 497
Belladonna, Essay on,............ 513
Baltimore Medical and Surgical Society............................. 723

Carsinoma, Excision of Rectum for............................. 17
Case illustrating the influence of Imagination upon the Voluntary Muscles.................. 20
Case of Bright's Disease of Mechanical Origin.....-............ 23
Case of Paroxysmal Hæmaturia, etc......................... 48
Chloral, Local use of............. 52
Choosing a Physician............ 55
Cæsarian Section, Successful case of............................. 56
Chronic Diarrhœa, Remedies for.................................. 60
Chrysophanic Acid,............... 63
Chloroform, Death from......... 119
Chronic Uterine Catarrh, Ergot in.................................. 127
Cinchonia Alkaloids and their Salts.............................. 155
Caseou's Degeneration.......... 235
Convention of American Medical Editors...................... 251
Croup, Remedies for.............. 256
Cincho-Quinine—What is it?... 264
Case of Successful Extirpation of the Parotid Gland.... 285
Case of Traumatic Tetanus in a young Child...................... 297
Colo-Rectitis......................... 332
Colchicum Root, Relative value of.................................. 349
Case of Potts' Disease of the Upper Cervical Vertebræ.:... 364
Cincho-Quinine as a Remedy, the importance of............... 369

Case of Stricture of the Urethra 388
Cincinnati Academy of Medicine 412
Charlatantry in Destructive Diseases 419
Chronic Aural Catarrh 449
Compend of Diagnosis in Paihological Anatomy 505
Cyclopædia of the Practice of Medicine 506, 561
Croup, Oldoina Treatment of 507
CAMPBELL, A. SIBLEY 541
CALHOUN, A. W. 641
Chicago Medical Society 725

Diploma Selling 56
DOUGHTY, W. H. 129
Department of Society Reports 372
Death after Chloroform 354, 511
Detroit Medical and Library Association 471
Death from Inhalation of Ether, 571
Diphtheria, Treatment of 638

Exchanges, Catalogue of 51
Editorial Correspondence 112, 115
190, 191, 251, 316, 373, 374, 436
439, 440, 504, 563, 564, 634, 635
703, 761
Esmarch's Operation for Closed Jaw 128
Erratum 319
Epistaxis, Simple Mode of Checking 320
European Correspondence 375
279, 441
Editorial Notes 560
Electro-Physiology 573, 609
Elements of Therapeutics 632
Excipients 637

Fever, Cerebro-Spinal 4
Free Phosphorus and the Phosphites 122
Fracture of the Patella 223

Glanders in the Human Subject, case of 228
GRANT, W. T. 393, 454, 523
Genu-Valgum, Operative Treatment of 414
Glyrite of Kephaline 422
Gonorrhœa 580
Guide to Therapeutics and the Practice of Medicine 631
GREENE, WM. A. 658
Georgia Pharmaceutical Association 759
GRIGGS, J. W. 705
Good Samaritan Hospital Medical Clinics 738

Hernia, Hypodermic Injection in 127
Hemorrhoidal Tumors, New Method of Treating 256
HENTZ, C. A. 275
Hypodermic Injections, Danger from 279
Hæmoptysis 358
How to Demonstrate the Presence of Carbonic Oxide in the air of a Room 384
Hospitals 447, 699
Hemorrhage from the Bowels 581
Hydrophobia, Cure for 640
Hog Cholera 743

Inebriety and Epilepsy, the Relation and Hereditary Tendency of 25
In Memoriam 47, 373, 701
Insufflation as an Auxiliary Method in Surgical Operations 126
Is Bromide Potassium and Sulph. Morphia an Antidote for Poisoning by Strychnine? 396
Introductory Lecture, etc 397
Illegitimate Advertising 627
Influence of Modern Education upon the Eye-Sight of Children 641

JOHNSON, J. T. 196, 536

Kerosene Lamps, 61
Kentucky State Medical Society, 127
KING, FERDINAND, 155, 200,
391, 593

Lister and the Edinburgh Students, 58
LOVE, WILLIAM ABRAM, 65
Lateral Movements in the Knee-Joints in cases of White Swelling 125
LOGAN, J. H. 147, 269
LEHARDY, J. C. 257

Medical Association of Georgia 47, 108, 700, 758
Medical Jurisprudence, a Contribution to 172

Maryland Medical Journal...... 189
*Mania a Potu*..................... 198
Micro-Photographs in Histology..........................255, 382
Meningitis.......................... 275
Moore, J. T........................ 396
Medical Colleges................... 436
Medical Societies.................. 504
Modern Medical Therapeutics and Modern Surgical Therapeutics.............................. 562
Medical Society of the District of Columbia....................... 628
Marrow Meatus — How Produced............................. 196
Medico-Chirurgical Society..... 715
Minutes of the Pharmaceutical Meeting........................... 717

Nervousness and Nervines...... 53
Notices.............................. 120
Nasal Catarrh....................... 257
Nephritic Colic..................... 391
Neuralgia, Nerve-Stretching in, 447
Nymphomania in a Mare Cured by Chloral......................... 512
Nicotinic Amaurosis.............. 541
Nitrate of Pitocarpia............ 685
Northern, Thos.................. 711
New York Therapeutical Society.............................. 719

Osteo-Cartilaginous Deposit in the Knee-Joint.................. 49
"Opium Antidote," Expose of.. 216
Opium Habit........................ 370
Opium in Hernia................... 658
Obstetrics, Interesting case of.. 417
Outlines of Modern Chemistry, 446
Otology, Recent Progress in.... 483
Obituary............................ 503
Outlines of Modern Chemistry, 633
Ophthalmoscope—How to use. 634
Opium Inebriety, the Responsibility of the Profession in Production of....................... 692

Pessary, New Intra-Uterine..... 1
Prolapsus Ani in Infants, Teatment of.......................... 18
Piles, Immediate Cure of........ 54
Pemberton, J. S.................... 71
Phosphorus—its use in Medicine.............................. 88
Pamplets Received,....191, 255, 382, 506, 760
Progressive Bulbar Paralysis, case of............................ 225
*Post Partem* Uterine Contractions, best Method of Promoting.............................. 294
Post Partum Hemorrhage...... 763
Pennsylvania Hospital Clinic.. 731
Puerperal Eclampsia........287, 705
Puerperal Insanity................ 321
Pathological Society of Philadelphia............................ 302
Pleuro-Pneumonia, etc........... 336
Proceedings of the Columbus, Ohio, Academy of Medicine.. 339
Plaster of Paris Jacket.......... 384
Phytolucca Decundra, etc... ... 477
Poisoning by Santonin........... 509
Paralysis of the Ocular Muscles, Orthopœdic Treatment of..... 639
Poison from Eating Ice Cream 658

Quinine, a Case of Idiosyncrasy in Regard to Effects of........ 36
Quinine in Diphtheria, Local Effects of......................... 640
Quackery in the Garb of Piety 570
Quinine.............................. 711

Reparative Surgery, Contribution to............................. 123
Rupture, Cure of.................. 192
Retention of Fractured Maxillæ 416
Relation of Blood to Vaccinal Immunity......................... 507
Retention of Urine, Treatment of.................................. 536

Stimulants used by the Race... 61
Sulphate of Cinchonidia and Sulphate of Quinine........... 65
Skin Diseases, Atlas of........... 121
Skin Diseases, Elementary Treaise on.......................... 122
Solution and Absorption of Medicines.......................... 123
Septicæmia after Amputations. 95
Subacute Pneumatic Arthritis.. 103
Stricture in the Male Urethra.. 193
Syphilis.............................. 200
Surgery, Recent Progress in... 230
Subaceous Tumors................ 256
St. Louis Medical Society....... 306
Sleeplessness, Remedies for... 320
Scott, D. W....................... 336
Suppurative Arthritis following Acute Rheumatism....... 352
Syphilology, Recent Progress in................................... 426
Simpson, C. A..................... 449
Saccharine Diabetes............. 475

Spermatorrhœa and Impotence 488
Skimmed Milk in Treatment of Catarrh of Bladder and Cystitis ... 496
Something New about Oxygen. 508
Supra-Pubic Lithotomy ... 528
STARR, E. F. ... 528, 581
Spinal Diseases, Plaster of Paris Jacket in ... 555
Strangulated Hernia, Successful Operation for ... 577
Sciatic Neuralgia ... 763

TALIAFERRO, V. H. ... 1, 321
The use of Water to Relieve Pain ... 53
Tax on Professions ... 111
Transactions of American Gynæcological Society ... 123
Transactions of the Pathological Society of Philadelphia.. 123
The New Chemical Nomenclature ... 147
The New Chemical Notation... 269
To Make Leeches Bite Promptly, ... 256
TAYLOR, W. A. ... 264
Trismus Nascentium ... 277
To our Contributors ... 314
To Physicians ... 320
Transactions of Medical and Surgical Society of Baltimore 337
Toledo Medical Association.... 410
Transactions of International Medical Congress of Philadelphia ... 505
Tapeworm, Treatment of ... 511
The Position of (Therapeutical) Damiana ... 512
TODD, J. S. ... 513
To our Subscribers ... 760
The Ear ... 633

Unethical Advertising ... 756
Uterine Cancer, Relief of Pain in ... 124
Uterine Diseases, Physic for... 186

Vaccination ... 760
Vesico-Vaginal Fistula, a Comparison of Operations for..... 50
Vaccination in Switzerland..... 59
Vesico Urethro-Vaginal Fistula, the Result of Venereal Ulceration ... 218
Viburnum Prunifolium ... 685

Wood Rendered Incombustible, 58
Water Supply of Augusta ... 129
WESTMORELAND, R. W. ... 193
WILSON, H. L. ... 198
WESTMORELAND, J. G. ... 332, 385, 397, 457, 590

Yellow Fever, Cause of ... 41

